# Grass species identity shapes communities of root and leaf fungi more than elevation

**DOI:** 10.1038/s43705-022-00107-6

**Published:** 2022-03-17

**Authors:** Stephanie N. Kivlin, Michael A. Mann, Joshua S. Lynn, Melanie R. Kazenel, D. Lee Taylor, Jennifer A. Rudgers

**Affiliations:** 1 Department of Ecology and Evolutionary Biology, University of Tennessee, Knoxville, TN 37996 USA; 2 Rocky Mountain Biological Laboratory, Crested Butte, CO 81224 USA; 3 Department of Biology, University of New Mexico, Albuquerque, NM 87114 USA

**Keywords:** Microbial ecology, Community ecology

## Abstract

Fungal symbionts can buffer plants from environmental extremes and may affect host capacities to acclimate, adapt, or redistribute under environmental change; however, the distributions of fungal symbionts along abiotic gradients are poorly described. Fungal mutualists should be the most beneficial in abiotically stressful environments, and the structure of networks of plant-fungal interactions likely shift along gradients, even when fungal community composition does not track environmental stress. We sampled 634 unique combinations of fungal endophytes and mycorrhizal fungi, grass species identities, and sampling locations from 66 sites across six replicate altitudinal gradients in the western Colorado Rocky Mountains. The diversity and composition of leaf endophytic, root endophytic, and arbuscular mycorrhizal (AM) fungal guilds and the overall abundance of fungal functional groups (pathogens, saprotrophs, mutualists) tracked grass host identity more closely than elevation. Network structures of root endophytes become more nested and less specialized at higher elevations, but network structures of other fungal guilds did not vary with elevation. Overall, grass species identity had overriding influence on the diversity and composition of above- and belowground fungal endophytes and AM fungi, despite large environmental variation. Therefore, in our system climate change may rarely directly affect fungal symbionts. Instead, fungal symbiont distributions will most likely track the range dynamics of host grasses.

## Introduction

Fungal symbionts colonize every plant species on Earth, often providing critical functions such as acquisition of soil water and nutrients [1], protection against pathogens [2] or herbivores [3], and mitigation of abiotic stress [4]. For example, fungal symbionts can increase plant biomass up to 120% under environmentally stressful conditions (reviewed by Kivlin et al. [5]), especially under thermal stress [6]. Consequently, plant-fungal interactions may shift realized plant niches compared to plants growing in isolation [7]. Fungi are a critical component of how plants cope with environmental stress under a changing environment [8]. While fungi may alter plant success in different environments, plants may in turn shape the composition of fungal communities, affecting the distribution of fungi along environmental gradients (reviewed by Rudgers et al. [9]). Therefore, determining how current abiotic factors structure the diversity and composition of plant-associated fungi can help to predict how plant-fungal associations will respond to future environmental change.

Despite the potentially critical roles of fungal symbionts in buffering plants from environmental extremes, their distributions along abiotic gradients, such as temperature gradients that track altitude, remain largely uncharacterized. Our meta-analysis [10] identified the knowledge gaps for altitudinal gradients, which are important because altitudinal range shifts have occurred for many plants as the climate has warmed [11]. These knowledge gaps include sparse data on root endophytes other than mycorrhizal fungi and a lack of studies that examine multiple fungal guilds (e.g., mycorrhizal fungi, root fungal endophytes, leaf fungal endophytes) across multiple plant species and replicated environmental gradients [10]. Most previous approaches have examined one environmental gradient [12–14], one plant species [15–18], or one fungal guild [19–21], hindering both generalizations about patterns of fungal symbiont distributions (see [10]) and the detection of environmental variables that underlie altitudinal patterns (see [22]).

At least three scenarios could occur as plant and fungal altitudinal ranges shift with climate change. 1: Plants and fungal symbionts could move up mountains in tandem. As long as the fitness outcomes of symbiosis are unchanged, overall plant-fungal interactions will remain stable. 2: Plants that migrate up mountains can gain novel fungal symbionts, including novel mutualists and novel pathogens. 3: Plants that migrate upward can lose fungal symbionts. Previous evidence for shifts in plant-fungal symbiosis along elevational gradients is mixed. While fungal mutualist abundance in plants can vary with elevation, the direction of the pattern appears to be highly specific to plant identity in the few studies that compare plant species across the same elevation gradients (e.g., [23, 24]). Likewise, limited empirical evidence for fungal symbiont diversity or composition turnover suggests both plant species identity and fungal guild identity could determine the patterns of fungal distributions along environmental gradients [10, 16, 25–28].

Aboveground and belowground fungal symbionts may face different environmental and plant host niche constraints that, along with dispersal capacities, influence their altitudinal distributions in divergent ways. For example, the soil environment may buffer root endophytes and arbuscular mycorrhizal (AM) fungi from thermal extremes in air temperatures. Aboveground, leaf endophytes may face greater thermal variation than root fungi, but less variation in other stressors, such as water or carbon resources. In deciduous plants, leaf endophytes must recolonize annually, whereas root endophytes may persist in roots and the soil matrix with fewer start-up costs. Finally, fungi that differ in host specificity may also diverge in sensitivity to environmental stressors. For example, in grasslands AM fungi are often host generalists ([29] but see [30]) that may have wide environmental tolerances, whereas pathogens can be highly specialized to hosts and environments [31]. Large-scale analysis of the specificity of leaf or root endophytes across the plant tree of life are lacking, but some evidence suggests that leaf endophyte composition may be strongly structured by plant species identity [20, 32] or plant traits [33, 34]. Root endophytes also vary in diversity and composition among plant taxa [35] and among root morphologies [36], but robust comparative analysis requires studies on multiple fungal guilds within the same plant taxa to parse plant identity from other variables.

Understanding shifts in fungal interaction network structures across niche space can advance general knowledge on fungal geographic distributions as well as inform predictions on future shifts in plant-fungal interactions under environmental change. Although the context-dependency of plant-fungal interactions remains an active area of investigation [9], to our knowledge, no studies have examined changes in both species and interaction turnover (i.e., shifts in network structure via changes in which fungal species are associated with which plant species) for fungal symbionts along replicated environmental gradients. Network structures of plants and fungi may shift along environmental gradients, even if overall community composition does not track environmental stress (e.g., for pollination: [37]). For example, rewiring could occur when all interacting species are present across locations, but a given species associates with either different partner species or different relative abundances of the same set of species in different environments. The symbiotic functions of fungi also vary with the environmental context [38]. For example, the same fungus may colonize one plant species in a warm, low elevation environment but associate with a different host species in a cold, high elevation environment. Plant-fungal interactions may also be specialized, such that a given set of fungi occupy only one environment or plant species [39]. Furthermore, fungal guilds may differ in average degree of specialization, reflecting their environmental niche, host niche, dispersal capability, or longevity. When environmental stress structures fungal communities, fungal and plant network structures may be least specialized at the highest, most stressful elevations due to abiotic factors overriding host interactions. Conversely, plant-fungal network structures may be more specialized at higher elevations when high elevation abiotic stressors magnify the costs/benefits of plant-fungal associations.

## Questions/hypotheses

To evaluate the relative importance of factors that structure leaf and root fungal symbiont communities, we sampled fungal endophytes from grasses across six elevation gradients that vary in abiotic conditions. We focus on grasses because grasses are common in mountain ecosystems and occur across large elevation ranges. Moreover, grass species host diverse fungal endophytes in their leaves in roots. We addressed the following questions.

1. What is the relative importance of environmental factors versus grass species identity for fungal symbiont diversity and community composition? Do fungal community patterns along environmental gradients differ among guilds: leaf endophytes, root endophytes, or arbuscular mycorrhizal fungi? We predicted grass identity to exceed environmental variables in structuring fungal symbiont communities along elevation gradients, and that fungal guilds differ in how community structure tracks environmental gradients, likely due to their location within the plant and primary functional roles. To evaluate these predictions, studies must sample the same plant taxa over replicated environmental gradients and characterize multiple fungal guilds within individual plants, components absent from prior studies.
2. Is there turnover in fungal symbiont identity or relative abundance with elevation, and how much does turnover vary among fungal guilds (i.e., leaf endophytes, root endophytes, AM fungi) or functional groups (i.e., pathogen, saprotroph, mutualist)? We predicted strong turnover with elevation, and that mutualistic/mycorrhizal fungi would dominate over saprotrophs and pathogens in the most extreme, highest elevation alpine environments.
3. Does grass-fungal network structure track elevation? If so, how much do fungal guilds differ in altitudinal variation in network structure? We predicted that network structure would track elevation most strongly for leaf endophytes among the fungal guilds because of their high exposure to climatic conditions that vary with elevation.

## Materials and methods

### Study sites

We sampled foliar fungal endophytes and root fungi (root endophytes and AM fungi) in the Colorado Rockies at the Rocky Mountain Biological Laboratory, Gunnison Co., Colorado, USA (38°57’N, 106°59’W). This region has predictable decreases in air temperature (*c*. 0.8 °C per 100 m; [40]) and declines in soil nutrients with altitude [41], but increases in precipitation, mainly as snow [42]. The entire region is warming at rates of 0.5–1.0 °C per decade [43].

To capture environmental, spatial, and grass-host specific variation in fungal guilds, we sampled 66 sites encompassing 9–13 elevations from each of six altitudinal gradients in July 2014 (Supplementary Table S1, Supplementary Fig. S1). Elevational gradients represented separate mountains in the Gunnison Basin and were located within 20 km of each other. We created a regional climate model to interpolate average number of growing degree days (GDD, base 0 °C), mean annual temperature (MAT), maximum temperature (Tmax), minimum temperature (Tmin), mean annual precipitation (MAP), and mean snow depth (MSD) for each site based on data from 29 local meteorological stations [44]. At each site, soil edaphic parameters were measured on dried soil at the UC Davis soils lab (see [24] for more details) and soil nutrients at Western Ag (Saskatoon, Canada). Soil pH was measured in a 1:1 solution with diH2O, and soil moisture was measured gravimetrically. Physical characteristics of each site (e.g., aspect, soil depth, elevation) were measured as described in Lynn et al. [44]. Environmental variation across sites was large. For example, MAT varied from 7.1 to 13.3 °C, MAP from 563 to 1171 mm, and Total N from 2 to 316 ug/g dry soil (Table S1).

### Host plant species

We focused on grasses because grasslands cover ~20% of Earth’s land surface [45] and dominate subalpine meadows of the Rocky Mountains. In addition, individual grass species spanned the entire elevational range of our study system [46], whereas tree, shrub, and forb species did not. At each location, we sampled nine adult individuals from up to 13 grass species representing five genera (Poaceae, subfamily Pooideae; Supplementary Table S1). Many sites had fewer than 13 grass species present, but all sites, except for two, had at least two grass species. Samples were composited by tissue type (leaves v. roots) and grass species within each site.

### Fungal composition

Collected root and leaf samples were surface sterilized (1 min in 95% ethanol, 2 min in 1% sodium hypochlorite solution, and 2 min in 70% ethanol) over ice to focus on the endophytic fungal community [34]. Following surface sterilization, samples were rinsed in purified water (Milli-Q Integral Water Purification System, EMD Millipore Corporation, Billerica, MA), stored in RNAlater, and refrigerated. All samples were then frozen in liquid nitrogen and ground using a mortar and pestle. Total DNA was extracted from ~50 mg of ground sample using QIAGEN DNeasy plant extraction kits (QIAGEN Inc., Valencia, CA).

Fungal composition was characterized using barcoded primers targeting the ITS2 region for leaf and root endophytes [47], and FLR3-FLR4 primers targeting ~300 bp in the 28S region for AMF [48]. Each PCR contained 5 μL of ~1–10 ng/μL DNA template, 21.5 μL of Platinum PCR SuperMix (Thermo Fisher Scientific Inc., Waltham, MA), 1.25 μL of each primer (10 μM), 1.25 μL of 20 mg/mL BSA, and 0.44 μL of 25 mM MgCl2. For the ITS2 primers, the reactions included an initial denaturing step at 96 °C for 2 min, followed by 24 cycles of 94 °C for 30 sec, 51 °C for 40 s, and 72 °C for 2 min, with a final extension at 72 °C for 10 min. For the 28S primers, reactions started with an initial denaturing step at 93 °C for 5 min, followed by 33 cycles of 93 °C for 1 min, 55 °C for 1 min, and 72 °C for 1 min, with a final extension at 72 °C for 10 min.

Three PCR replicates from each sample were pooled and then cleaned and concentrated using a ZR-96 DNA Clean & Concentrator-5 (Zymo Research Corporation, Irvine, CA). PCR was then carried out on all samples to add dual indexes and Illumina sequencing adaptors; each reaction began with an initial denaturing step at 98 °C for 30 s, followed by 7 cycles of 98 °C for 30 s, 62 °C for 30 s, and 72 °C for 30 s, with a final extension at 72 °C for 5 min. Sequencing was performed by the Genomic Sequencing and Analysis Facility at The University of Texas at Austin using paired-end 250 base Illumina MiSeq v.3 chemistry (Illumina, Inc., San Diego, CA). We aimed to obtain a minimum of 30,000 reads/sample for the ITS2 region and 20,000 reads/sample for the 28S region. All sequences are deposited in the NCBI SRA database under accession number (PRJNA639093).

### Bioinformatics

We processed reads to generate OTUs using commands from USEARCH (v9.2.64). Reads from previous studies [24] and this study were clustered together to improve OTU delineations for a total of 36,754,931 reads. We merged paired-end reads using the fastq_mergepairs from USEARCH with “fastq_maxdiffs” set to 20 and “fastq_maxdiffpct” set to 10 to ensure proper merging at a low error rate. The merged reads and the forward unmerged reads were trimmed at the primer sites using cutadapt with “e” set to 0.2, “m” set to 200, and untrimmed reads were discarded. Merged reads were filtered using fastq_filter from USEARCH with “fastq_maxee” set to 1.0. The forward reads were first trimmed to 230 using fastx_truncate from USEARCH with “trunclen” set to 230 and then filtered by fastq_filter from USEARCH with “fastq_maxee” set to 1.0. We then concatenated the merged and forward reads into one file and de-replicated using fastx_uniques from USEARCH with “minuniquesize” set to 2. After these steps, 11,357,274 sequences remained. We clustered these sequences to form OTUs at 97% similarity [49] using cluster_otus command from UPARSE. The reads (all reads before filtering step) of each sample were mapped to OTUs with usearch_global from USEARCH with “id” set to 0.97. We determined taxonomy for the representative OTUs using sintax from USEARCH with the database set to UNITE all eukaryotes (v. 8.2) “strand” set to both and “sintax_cutoff” set to 0.8 [50]. Representative OTUs were also blasted against Genbank with “perc_identity” set to 80 and “max_target_seqs” set to 50. All OTUs identified as “fungi” were retained, and OTUs labeled as “unknown” or “unidentified” were manually inspected based on blast results to determine retention. Our filtering criteria left between 5 and 418 OTUs per sample (Supplementary Table S2).

Due to low fungal abundance in leaves [34], many leaf samples were dominated by plant sequences (average ~78% plant reads). Therefore, fungal sequence numbers in leaf samples were low, despite adequate sequencing depth to capture trends in fungal endophyte communities across sites based on prior analyses [24, 34, 35]. We included only samples that contained at least 50 fungal sequences after data processing (Leaves *N* = 192, Roots *N* = 191, AMF *N* = 251), and most samples had much greater sequencing depth, especially for roots (Supplementary Table S2). Nevertheless, there were no correlations between sequence read depth and richness, alpha diversity, or evenness of our samples (*P* > 0.05 in all cases), and plant species did not differ in the average sequencing depth for samples (*P* > 0.05). Data for each fungal OTU were transformed to the proportion of total sequence abundance to minimize any differences in sampling effort [51].

#### Diversity and composition

We calculated the alpha diversity metrics of richness, Shannon’s Diversity, Inverse Simpson’s Diversity, and Pielou’s Evenness. For each fungal guild, differences among plant species and elevation in alpha diversity were first determined using a general linear mixed effects model with plant species (categorical) and elevation (continuous) as fixed effects and site nested within elevation gradient (e.g., mountain identity, Supplementary Table S1, Supplementary Fig. S1) as random effects to account for the lack of statistical independence among plant species sampled at the same site and among sites located within the same mountain elevation gradient (Supplementary Fig. S1). Models were constructed using the lmer function in R package lme4 [52, 53]. To address, *do fungal community patterns along environmental gradients differ among guilds: leaf endophytes, root endophytes, or arbuscular mycorrhizal fungi?*, we then compared alpha diversity metrics among fungal guilds using a general linear mixed effects model with fungal guild, plant species, and elevation as fixed effects and site nested within elevation gradient as random effects. In all models, we evaluated parameter fit with analysis of deviance using Wald chi-square tests and corrected for multiple comparisons using a false discovery alpha of 0.05. Differences among grass species were determined using Tukey post-hoc tests.

Because elevation is a good proxy for variation in both climate and soil parameters (Supplementary Table S1), in all community analyses, we first ran models with grass species and elevation to parse biotic versus abiotic influences on fungal OTUs, then secondly ran full variance partitioning models with all environmental covariates (Supplementary Table S1, climate, physical, soil) in addition to grass species identity and space (gradient location, Supplementary Fig. S1). Because leaf and root endophytes were sequenced using different primers than AM fungi, we could not compare composition among the three guilds directly. Instead, we compared the relative influence of biotic and abiotic drivers on fungal composition within each guild to compare patterns among guilds. To do so, we first used distance-based redundancy analysis (dbRDA) to analyze the effects of plant host species and elevation on fungal composition for general fungal communities in leaves and roots and separately for AM fungal communities in roots. All models were run on quantitative Jaccard indices of fungal composition for each guild and included site nested within elevation gradient (e.g., mountain side, Supplementary Fig. S1) as random effects. Second, to evaluate which environmental variables most strongly influenced fungal composition, we further partitioned variance in fungal composition due to grass species, climate variables (MAP, MAT, MSD, Tmax, Tmin, and GDD), soil variables (total nitrogen, total phosphorus, nitrate, ammonium, calcium, magnesium, potassium, iron, manganese, sulfur, aluminum, soil pH, soil gravimetric moisture content), physical variables (aspect degree, aspect category (e.g., cardinal direction), slope, soil depth, and elevation) and spatial variables (latitude and longitude) using the varpart function in Vegan v. 2–5.3 [54]. Plots of fungal composition by plant host were also generated using dbRDA separately for each fungal guild. Spatial variables were de-trended and tested for spatial autocorrelation using the ade4 package v. 1.7–16 [55]. When we detected significant spatial autocorrelation eigenvectors, we included these in the spatial variable matrix. To characterize how many fungal taxa occurred in multiple plant taxa and elevations, we used the VennDiagram package v. 1.6.20 [56].

#### Turnover and rewiring

To evaluate whether fungal composition was driven by grasses associating with different fungal taxa or differing relative abundances of the same fungal taxa, we first performed a beta partitioning analysis using betapart v. 1.5.3 [57]. Each fungal guild was analyzed separately. Next, to examine turnover in the abundances of fungal functional groups (pathogens, saprotrophs, mutualists), we defined groups using the FungalTrait database, which merges previous databases into one cohesive framework of 17 functional trait types (referred to here as functional groups; [58]). We recognize that fungal functions are highly environmentally dependent and therefore these functional groups may represent potential function more than actual function. Functional group identity was ascribed to 60% of leaf endophyte and 62% of root endophyte fungal taxa. Then, cumulative abundance of proportionally transformed sequence reads in each functional group was analyzed using a general linear mixed effects model with grass species and elevation as fixed effects and site nested within elevation gradient as random effects, as above. Finally, we defined indicator species within the OTUs that comprised at least 1% of the total abundance of each fungal guild by grass host, gradient, and elevation classes (rounded to the nearest 100 m) using the indicspecies package v. 1.7.9 [59]. Functional group assignments using the FungalTrait database from above were assigned to each indicator taxon [58]. A large percentage of significant indicator taxa out of the total number of OTUs would confirm that turnover in the species identity of fungal associations is stronger than turnover in the relative abundances of the same fungal taxa.

#### Network properties

To address *does grass-fungal network structure track elevation?*, we analyzed four properties that encompass different facets of ecological networks at the site level. First, we calculated network *nestedness*, or the propensity for specialists to interact with the same plant species as generalists, using the weighted NODF (Nestedness metric based on Overlap and Decreasing Fill; [60]). Second, we calculated *complexity* as linkage density or the average number of interactions per plant species [61]. Third, to characterize *specialization*, we used the *H2’* Index [62]. Finally, network *evenness* was calculated as Alatalo’s interaction evenness [63]. In all cases, these network metrics were weighted indices to increase accuracy [64], and calculations were performed in the Bipartite package v. 2.15 [65]. To address, *how much do fungal guilds differ in altitudinal variation in network structure?*, we compared network-level statistics among fungal guilds using a general linear mixed effects model with fungal guild as a fixed effect, number of grass hosts as a fixed effect, and gradient as a random effect (function lmer in lme4 [52],). We compared relationships with elevation separately for each fungal guild, using general linear mixed effects models with elevation as a fixed, continuous effect, number of grass hosts within the network as a fixed, continuous effect, and gradient identity as a random effect (Supplementary Table S1, Supplementary Fig. S2). We evaluated parameter fit with analysis of deviance using Wald chi-square tests using the car package 3.0–10 in R [66].

All data met model assumptions of normality of residuals and homogeneity of variance. All analyses were performed in R v. 3.5.0 [53].

## Results

*1: What is the relative importance of environmental factors versus grass species identity for fungal symbiont diversity and community composition? Do patterns along environmental gradients differ among fungal guilds: leaf endophytes, root endophytes, or arbuscular mycorrhizal fungi?*

### Alpha diversity

**Fig. 1:**
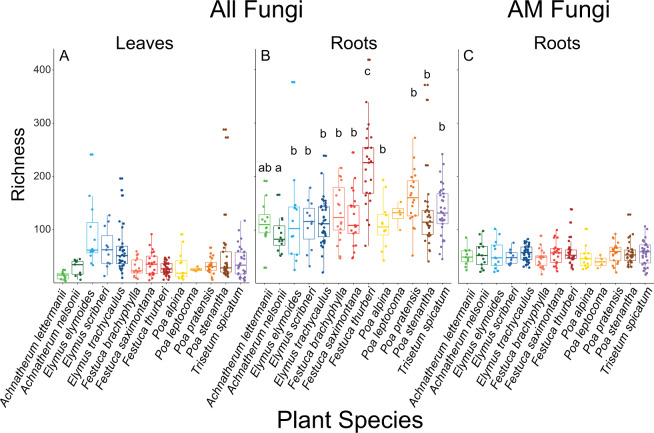
Fungal richness by grass species. Richness by grass species for leaf fungal endophytes (**A**), root fungal endophytes (**B**), and AM fungi (**C**). Means and 95% confidence intervals are plotted for each grass species. Tukey posthoc designations after correcting for false discovery rate of alpha = 0.05 are denoted with lower case letters. Grass species always contributed more to the variation in fungal alpha diversity than elevation.

Grass identity was more important to fungal symbiont diversity than elevation. Leaf endophyte richness (χ^2^ = 39.8, *P* < 0.001; Fig. 1A), Shannon’s Diversity (χ^2^ = 94.4, *P* < 0.001; Supplementary Fig. S2A), Inverse Simpson’s Diversity (χ^2^ = 30.5, *P* = 0.002; Supplementary Fig. S2D), and Pielou’s Evenness (χ^2^ = 90.5, *P* < 0.001; Supplementary Fig. S2G) differed among grass species but not elevations. Similarly, grass species explained the most variation in root endophyte richness (χ^2^ = 92.2, *P* < 0.001; Fig. 1B), Shannon’s Diversity (χ^2^ = 51.5, *P* < 0.001, Supplementary Fig. S2B), Inverse Simpson’s Diversity (χ^2^ = 30.2, *P* = 0.003; Supplementary Fig. S2E), and Pielou’s Evenness (χ^2^ = 24.5, *P* = 0.017; Supplementary Fig. S2H), and no root endophyte diversity indices varied significantly with elevation. AM fungal diversity also only differed among grass species but only for Shannon’s Diversity (χ^2^ = 24.5, *P* = 0.018; Supplementary Fig. S2C) and Inverse Simpson’s Diversity (χ^2^ = 33.1, *P* = 0.001; Supplementary Fig. S2F) indices.

Fungal symbiont guilds differed in mean diversity, despite a lack of altitudinal trends in diversity. The root endophyte guild was the most diverse, and the leaf endophyte guild was the least diverse. Specifically, root endophyte richness (mean = 137) was ~2.5x larger than either leaf endophyte (mean richness = 52) or AM fungi richness (mean richness = 55, *P* < 0.001). Similarly, Shannon’s Diversity of root endophytes was ~1.2x higher than leaf endophytes and ~1.3x higher than AM fungi (*P* < 0.001). Also, the Inverse Simpson’s Diversity of root endophytic fungi was higher than AM fungi and leaf endophytic fungi (*P* < 0.001). Evenness was ~1.1x higher for AM fungi than for leaf or root endophyte guilds (*P* < 0.001).

### Composition

**Fig. 2:**
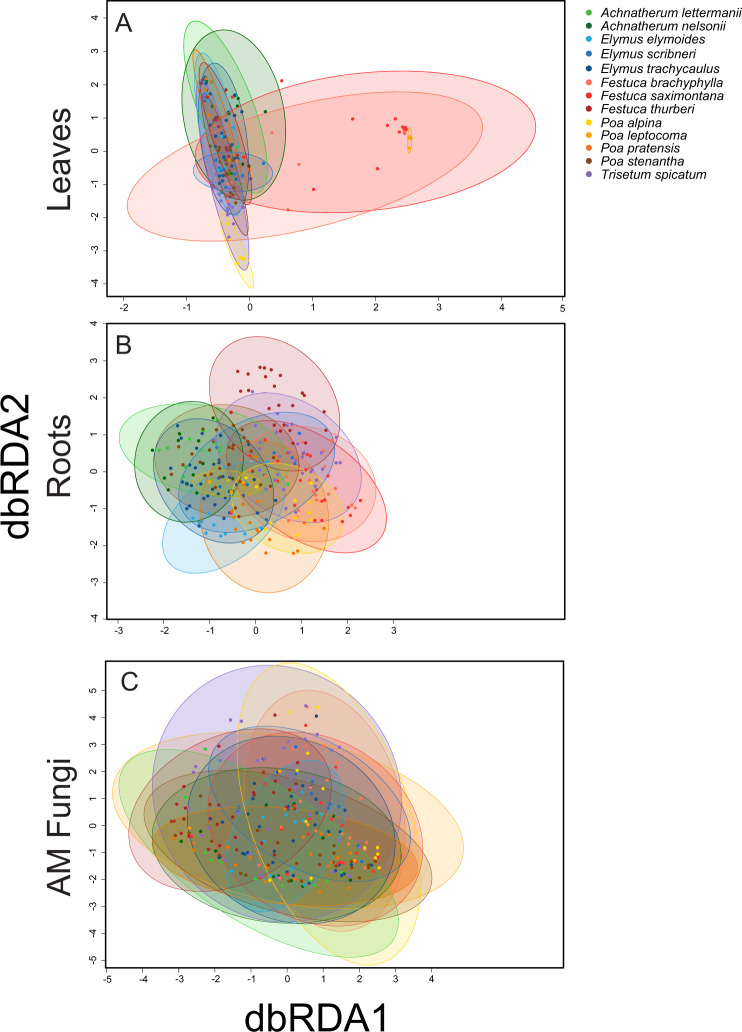
Fungal community composition by grass species. dbRDA ordination for 13 grass species for leaf endophytes (**A**), root endophytes (**B**), and AM fungi (**C**). Points represent the composition of a given sample and ellipses are the standard error of fungal composition for each grass species. The differences between leaf and root endophyte communities was largest for *P. stenantha* and *T. spicatum*, and smallest for *P. leptocoma*. All fungal guilds differed in fungal community composition among plant species (*P* < 0.05).

**Fig. 3:**
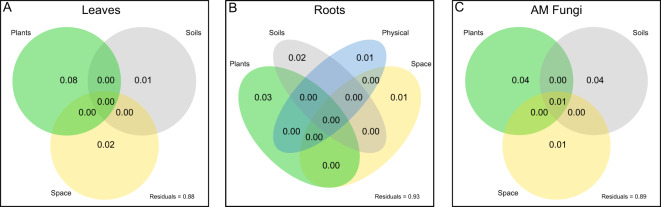
Variance partitioning of drivers of fungal community composition. Variance partitioning of leaf (**A**) and root (**B**) fungal endophytes and AM fungi (**C**) among all statistically significant plant, soil, climate, physical and spatial environmental variables. Numbers in the Venn diagram represent adjusted R squared values of variance explained by each factor alone and in combination with other factors in the model. Climatic variables never explained significant variation in fungal composition.

Fungal guilds also diverged in the relative importance of grass species identity and elevation for explaining variation in community composition. Leaf fungal endophyte communities varied only by grass species (χ^2^ = 285.0, *P* < 0.001; Fig. 2A, Supplementary Fig. S3) but not by elevation (*P* > 0.05). Variance partitioning analysis upheld grass species as the main driver of variation in leaf fungal endophyte composition (adj*R*^*2*^ = 0.08, *P* < 0.001; Fig. 3A), with ancillary, small contributions from soil factors (adj*R*^*2*^ = 0.01, *P* < 0.001) and spatial geography (adj*R*^*2*^ = 0.02, *P* = 0.018). In contrast, for root endophytes, both grass host identity (χ^2^ = 109.1, *P* < 0.001; Fig. 2B, Supplementary Fig. S3) and elevation (χ^2^ = 21.7, *P* < 0.001) explained variation in composition. In the full model of variance partitioning, grass identity still contributed the most to root fungal endophyte composition (adj*R*^*2*^ = 0.03, *P* < 0.001), followed by soil factors (adj*R*^*2*^ = 0.02, *P* < 0.001), and a small influence of spatial geography (adj*R*^*2*^ = 0.01, *P* < 0.001; Fig. 3B). For AM fungi, grass species also explained more variance in community composition (χ^2^ = 54.7, *P* < 0.001) than elevation (*P* > 0.050) and was the primary driver of AM fungal composition (adj*R*^*2*^ = 0.05, *P* < 0.001; Fig. 2C, Supplementary Fig. S3) when compared against all environmental variables. Like root endophytes, AM fungi composition tracked soil variables (adj*R*^*2*^ = 0.05, *P* < 0.001) and spatial geography (adj*R*^*2*^ = 0.02, *P* = 0.007; Fig. 3C), but these factors explained more variation in the composition of AM fungi than in the composition of leaf or root endophytes.

*2: Is there turnover in fungal symbiont identity or relative abundance with elevation, and how much does turnover vary among fungal guilds or functional groups?*

**Fig. 4:**
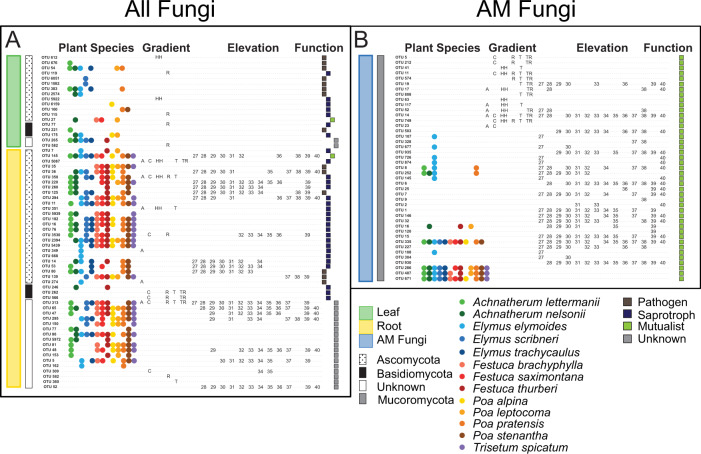
Indicator fungal taxa. Indicator taxa by tissue type for (**A**) leaf endophytes (green bar), root endophytes (yellow bar), or (**B**) AM fungi (blue bar) including phylum for each fungal taxon. Significant indicator taxa are coded by plant species they colonize (circles), elevational gradient (letter designations) or elevation class (number designations rounded to the nearest 100 m). Each indicator species was color coded by functional group using squares: pathogen (brown), saprotroph (dark blue), mutualist (green), or unknown (gray). Root endophytes tended to preferentially colonize certain plant species (38/45 indicator taxa; 84%) as did leaf endophytes (12/18 indicator taxa; 67%). AM fungi instead were mostly associated with different elevations (30/43 indicator taxa; 70%).

Variation in composition within fungal guilds was always due to turnover in fungal species identity (leaves = 99%, roots = 99%, AM fungi = 98.8%), rather than changes in the relative abundance of the same fungal taxa (all <0.01%). Indicator taxa confirmed these trends because we identified a large percentage of indicator taxa, especially for AM fungi (1% leaf fungal endophyte taxa, 2% root fungal endophyte taxa, 14% of AM fungal taxa; Supplementary Table S3), a sign of high turnover. We predicted the most altitudinal turnover for leaf endophytes because of their exposure to air temperatures that vary strongly with elevation [22]. Unexpectedly, leaf indicator taxa grouped mostly by grass host (84%; Fig. 4A), with no signal from elevation. For root endophytes, indicator taxa mostly grouped by grass host (67%) with some influence of elevation (49%; Fig. 4A). In contrast, AM fungi indicator taxa grouped mostly by elevation (70%), with smaller influences of grass species identity and spatial location (Fig. 4B).

**Fig. 5:**
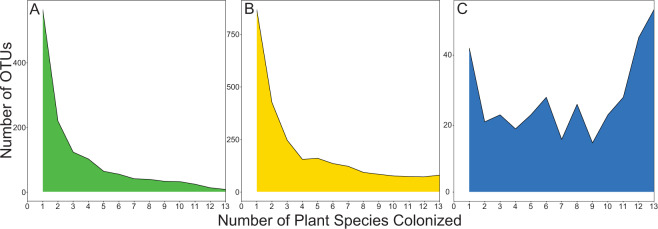
Plant host breadth for each fungal OTU. Number of plant species colonized by each leaf fungal endophyte OTU(**A**), root fungal endophyte OTU(**B**) or AM fungal OTU (**C**). Leaf and root endophytes mostly colonized only one plant species, but AM fungi typically colonized more than one plant species (median host species = 8), with 15% of AM fungal OTUs colonizing all plant species.

Most root endophyte indicator taxa were putative saprotrophs (49%), and most leaf endophyte indicator taxa were putative pathogens (44%); all AM fungal indicator species were by definition mutualists due to the trait database designation. Due to the strong signal of grass host influence on both composition and indicator taxa, we examined how many host species each fungal taxon colonized. When all OTUs were considered, many leaf endophyte taxa (43%) and root endophyte taxa (34%) colonized only one grass species (Fig. 5A,B), while less than 1% of leaf endophyte taxa and 1% of root endophyte taxa occurred in all 13 grass species. However, AM fungi were more generalist, with 15% of taxa colonizing all grass species (Fig. 5C, median number of host taxa = 8), and only 12% colonizing one grass host. However, host specificity may be highest among OTUs that are relatively rare. When we considered only OTUs that are comprised of at least 1% of overall sequence reads, 14% of leaf endophyte taxa, 4% of root endophyte taxa and less than 1% of AM fungal taxa colonized only one grass species. Conversely, among these abundant OTUs, 1% of leaf endophyte taxa, 11% of root endophyte taxa and 26% of AM fungal taxa colonized all grass species.

Plant specificity even extended to fungal functional groups. Shifts in fungal functional group composition (pathogens, saprotrophs, and mutualists) tracked grass identity for both leaf endophytes (Pathogens χ^2^ = 31.2, *P* = 0.002; Saprotrophs χ^2^ = 51.2, *P* < 0.001; Mutualists χ^2^ = 204.9, *P* < 0.001), and root endophytes (Pathogens χ^2^ = 36.4, *P* < 0.001; Saprotrophs χ^2^ = 26.6, *P* = 0.009; Mutualists χ^2^ = 77.6, *P* < 0.001; Supporting Information Fig. S4) with no significant influence of elevation (*P* > 0.050).

*3: Does grass-fungal network structure track elevation? If so, how much do fungal guilds differ in altitudinal variation in network structure?*

**Fig. 6:**
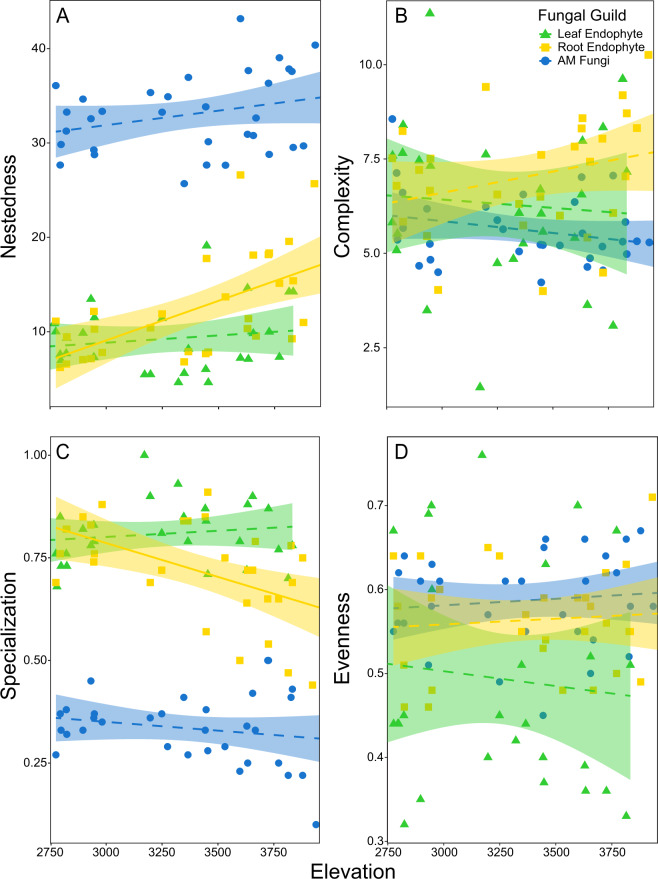
Network indices across elevations for leaf endophytes (green triangles), root endophytes (yellow squares) and AM fungi (blue circles). In all cases, solid lines indicate significant relationships with elevation at (*P* < 0.05) whereas dashed lines were insignificant (*P* > 0.05), bands are 95% confidence intervals. AM fungal networks were consistently less specialized (H2’) and more nested (Nestedness metric based on overlap and decreasing fill; NODF) than either leaf (*P* < 0.001) or root endophyte networks (*P* < 0.001, **A** and **B**). Only root endophyte network structure tracked elevation; fungi in roots were both less specialized (H2’, *P* < 0.001) and more nested (NODF, *P* < 0.001) at higher elevations. Complexity, as measured by linkage density, and interaction evenness did not vary among fungal guilds or with elevation (all *P* > 0.05; **C** and **D**).

Grass-fungal network structure tracked elevation only for the root endophyte guild. For root endophytes, network nestedness increased at higher elevations (χ^2^ = 14.9, *P* < 0.001; Fig. 6A) whereas network specialization decreased at higher elevations (χ^2^ = 11.8, *P* < 0.001; Fig. 6B). AM fungi and leaf endophyte network structure did not significantly track elevation (*P* > 0.050, Fig. 6). None of the fungal guilds had network complexity or evenness metrics that followed elevation gradients (*P* > 0.050; Fig. 6C, D).

Regardless of elevation, network nestedness (χ^2^ = 17.371, *P* < 0.001) and specialization (χ^2^ = 16.571, *P* < 0.001) significantly varied among the fungal guilds. AM fungi networks were the least specialized and most nested, whereas leaf endophyte networks were the most specialized and least nested, and root endophyte networks had intermediate network properties. All fungal guilds differed from one another in network nestedness and specialization (*P* < 0.05).

## Discussion

Predicting how plant-fungal associations will respond to future environmental change requires understanding how current environments structure fungal symbiont diversity and composition. Here, differentiation of fungal communities among grass species in the Rocky Mountains of Colorado was unexpectedly high given both the large environmental heterogeneity encompassed by our 2700–4100 m span in elevation and the small spatial scales at which grass species co-occurred within sites (generally 0.1–10 m). Indeed, metrics of alpha diversity and composition for all fungal guilds varied more among grass species than with other environmental drivers, including elevation or spatial geography (e.g., identity of the mountain sampled). Plant identity was a surprisingly strong structuring force given the constraint of our study to a single subfamily of grasses. If plants are the main driver of symbiotic fungal community structure, then future environmental changes, such as climate warming or nitrogen deposition, are likely to have the largest impacts on symbioses through shifts in plant species geographic ranges, population sizes, or phenologies, rather than through the direct effects of temperature, eutrophication, or other abiotic drivers. Many studies have documented shifts in plant species distributions [11] or phenologies [67] associated with environmental change, yet it remains unknown how much plant interactions with fungal symbionts (or other species) will be impacted by these shifts [44, 68]. Given the high plant-specificity in our study, if dispersal trajectories or phenologies of plant and fungal partners shift at different rates, it is likely that plant-fungal interactions will re-assort with changing environmental conditions, a phenomenon named *re-ordering* (see [9]).

Previous studies along the same altitudinal gradients revealed that culturable leaf fungal endophytes [34] and root fungal endophytes [35] were also strongly differentiated among plant taxa. Similarly, a survey of three of the grass species included here using next-generation sequencing methods at half of our sites including an earlier time period (year 2012) confirmed a strong influence of grass species identity on fungal endophyte composition that exceeded the impacts of long-term, field experimental warming [24]. Differences in fungal endophyte identity among plant hosts may occur because of differences in plant size [18], plant chemistry [69], plant habitat, or other plant traits [70]. Here, because the grass species we surveyed had similar habitats and also similar phenologies that track snow melt [44], habitat and phenology were unlikely to be important drivers. Plants are the proximate habitat for the fungal endophytes and mycorrhizal fungi surveyed here and as such may buffer these fungi from external climatic or resource stressors (e.g., freezing temperatures, carbon resources). Indeed, many studies have demonstrated differences in plant-associated microbiomes among plant hosts (e.g., [71–73]). However, most previous studies either combine data across multiple studies (confounding plant species identity with location/environment) or focus on only one fungal guild or plant species along one environmental gradient, limiting the ability to parse environmental variables as sources of variation in community structure. Here, we demonstrate robustly that plant species conditioning of fungal endophytic and mycorrhizal diversity and composition was consistent across 13 grass species and six steep environmental gradients in our regional survey of the Rocky Mountains of North America.

Although grass identity had an overriding influence on all fungal guilds, leaf fungal diversity and composition were most strongly influenced by grass species identity, while AM fungi were least influenced by their plant host association. Leaf endophytes may form tight associations with plants by continuously living in plant meristems [74] or nearby leaf litter [75], even during dormancy under nine months of snowpack, and may also be more dominated by host-specific pathogens than root-associated communities, as suggested by our survey. Conversely, root endophytes and particularly AM fungi may require generalist strategies, colonizing multiple nearby plant hosts via common fungal networks belowground [76] or may experience common environmental filtering processes in soils versus plant hosts (e.g., [77]).

Despite advances in analyses during the past few decades (e.g., [33, 78]), the ecological specificity of most plant-associated fungal taxa is still unknown [30]. Here, plant pathogens were the most likely to be host-specific in grass leaves while saprotrophs represented many of the root endophyte indicator taxa. Indicator AM fungi in contrast differed more among elevations or mountainsides than did root or leaf endophytes. These results were especially evident at the taxon level; many leaf and root endophyte taxa associated with only one host, whereas many AM fungal taxa associated with all 13 grass species. These results were further supported by the fact that turnover in fungal taxa was high in all fungal guilds, with few examples of changes in abundance of the same taxa. A previous study similarly demonstrated that root endophytes had more plant-host specific associations than AM fungi [13]. Similarly, plant-pathogen relationships are often more host-specific than other fungal functional types (reviewed by Barrett et al. [79]), especially in long-lived plants.

At broad levels of biological organization, fungal functional groups demonstrated greater specificity among grass hosts than structuring by elevation, similar to the patterns for specific fungal taxa. Although fungal symbionts can buffer plants from environmental stress in experimental trials (reviewed by Kivlin et al. [5]), our results suggest that the costs and benefits of fungi may be highly host specific. Here, along steep environmental gradients, some grass species (e.g., *Achnatherum lettermannii*) hosted more putative pathogens while others (e.g., *Poa leptocoma*) hosted more putative mutualists. Given the role of beneficial fungi in plant stress amelioration [5], we expected mutualists to dominate more stressful, high elevation environments, but our evidence indicates that such relationships strongly depend on grass host identity. A caveat is that all grass species were not found across all elevations in our survey. However, our results at the grass-species scale (here and in [24]), combined with previous tests of variation in grass-species-specific fungal associations across environmental gradients support the conclusion that environmental trends in the abundance of putatively mutualistic fungi are highly host-specific [10, 26, 28]. A possible explanation for this host plant-specificity is that grass species were sampled across different subsets of their full geographic distributions [44]. Expansion of data collection efforts into lowlands and over additional axes of plants’ latitudinal distributions could improve resolution (e.g., [80]).

Plant-fungal network analysis provides important information on gradients in species interactions that cannot be detected through traditional analyses of composition and diversity (e.g., [19]). In our study, metrics of fungal network structure were not affected by which grass species were present, despite the strong signal of grass identity on fungal diversity and composition. Instead, network properties varied most strongly among fungal guilds rather than with elevation or grass species composition. AM fungal networks were the most nested and the least specialized, whereas leaf endophyte networks were the most specialized and least nested. Root endophyte networks were the only networks in which properties tracked elevation, with lower specialization at higher elevations. We expected all fungal networks to become more specialized at higher elevation sites, if high elevation environmental stressors structured the costs/benefits of plant-fungal associations. However, specialization may decline with elevation if fungi become more strongly filtered by the abiotic environment than by the host plant. AM fungi appeared robustly general in their grass host associations, and leaf fungal endophytes too host specific for network properties to track the large environmental variation along elevation gradients. Other studies of fungal symbiont networks across elevations are rare. However, one prior study reported that leaf fungal endophytes were most specialized at intermediate elevations, but only examined one tree species on one elevation gradient [19]. Altogether, there is no support to date that elevation-induced climatic stress gradients structure entire plant-fungal networks.

To our knowledge, our study is the most comprehensive survey of plant-associated fungi along elevation gradients to date (reviewed by Kivlin et al. [10]); however, our work had some limitations. First, although all grass species that we surveyed occurred in at least three elevations bands along each replicated elevation gradient (e.g., mountainside), only 3 of 13 plant species (*Elymus trachycaulus, Poa stenantha, Trisetum spicatum*) occurred across all elevations that we sampled. In previous studies, we demonstrated that a plant’s elevational range distribution did not influence fungal endophyte composition in leaves [34] or roots [24]. Nevertheless, we could not test for the interaction of elevation and grass species due to the limitation that not all species were present across all elevations. Second, the change in grass composition with elevation affected how many grass hosts were included in bipartite networks constructed at each elevation. However, we tested for this limitation by bootstrapping network statistics to only include five grass species per site, and we did not detect any influence of which grass taxa were included on the final network statistic in any case. Leaf endophyte colonization rates and abundance were low in our sites [34], decreasing DNA yields from this tissue type (Supplementary Table S2), which could influence conclusions, but likely represents biological differences in their abundances relative to root fungi. Primer specificity among fungal guilds necessitated that we target the ITS2 region for foliar and root endophytes and the more conserved LSU region for AM fungi. Therefore, comparisons of richness and diversity among guilds should be interpreted in light of the fact that less conserved regions detect greater diversity among sequences. Finally, our study was restricted to long-lived, perennial C_3_ grasses, which makes the conclusion of an overriding influence of plant identity conservative because we expect much larger host-specificity of fungal symbionts when comparing e.g., trees versus grasses. However, the degree to which the patterns of plant host influence extend to other plant species with different life histories, elevation ranges, or traits remains to be determined.

## Conclusion

Environmental change has the potential to either directly affect plant-associated fungi or indirectly alter these fungi via their interactions with plant hosts [8]. Here we demonstrated robust plant host structuring of leaf endophytes, root endophytes, and AM fungi across 13 co-occurring grass species along replicated elevation gradients. Grass identity explained the most variation in plant-associated fungal diversity and composition, despite large abiotic variation among sampling locations. Therefore, as climates change, plant-fungal associations may only be affected if plant species shift their geographic distributions [9, 81]. If plant-associated fungi are unable to keep up with these spatial shifts in plant distributions due to dispersal limitations, then current plant-fungal symbioses may become decoupled. Our data demonstrate that this decoupling could occur in all fungal guilds but is most likely in leaf-associated fungal endophytes, which were most strongly structured by grass identity.

## Supplementary information

Figure S1

Figure S2

Figure S3

Figure S4

Table S1

Table S2

Table S3

## Data Availability

Sequencing data is archived in GenBank. All other data is archived in EDI.
