# Supplementary figures and images for: Grass species identity shapes communities of root and leaf fungi more than elevation
Source: ISME Commun. 2022 Mar 17;2:25. doi: 10.1038/s43705-022-00107-6 (PMC9723685; doi:10.1038/s43705-022-00107-6)

**Figure S1.** Map of study sites color coded by elevation.


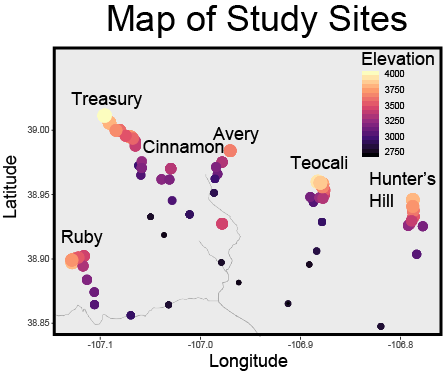

Supplement: Supplementary file 1 — Figure S1 [file 43705_2022_107_MOESM1_ESM.docx]
