# Supplementary material for: Grass species identity shapes communities of root and leaf fungi more than elevation
Source: ISME Commun. 2022 Mar 17;2:25. doi: 10.1038/s43705-022-00107-6 (PMC9723685; doi:10.1038/s43705-022-00107-6)
Supplement: Supplementary file 2 — Figure S2 [file 43705_2022_107_MOESM2_ESM.docx]

Figure **S2**. Shannon’s Diversity (A-C), Inverse Simpson’s Diversity (D-F) and Pielou’s Evenness (G-I) indices by grass species for leaf fungal endophytes (A, D, G), root fungal endophytes (B, E, H), and AM fungi (C, F, I). Means and 95% confidence intervals are plotted for each grass species. Grass species identity always contributed more to the variation in fungal alpha diversity than elevation. Tukey posthoc designations after correcting for false discovery rate of alpha = 0.05 are denoted with lower case letters.
