# Supplementary material for: Grass species identity shapes communities of root and leaf fungi more than elevation
Source: ISME Commun. 2022 Mar 17;2:25. doi: 10.1038/s43705-022-00107-6 (PMC9723685; doi:10.1038/s43705-022-00107-6)

Figure **S3**. dbRDA ordination for 13 grass species, separated by genus but retaining the same dbRDA axis values, for leaf endophytes (A-F), root endophytes (G-L), and AM fungi (M-R). Points represent the composition of a given sample and ellipses are the standard error of fungal composition for each grass species. The differences between leaf and root endophyte communities was largest for *P. stenantha* and *T. spicatum*, and smallest for *P. leptocoma*. All fungal guilds differed in fungal community composition among grass species (*P* < 0.05).


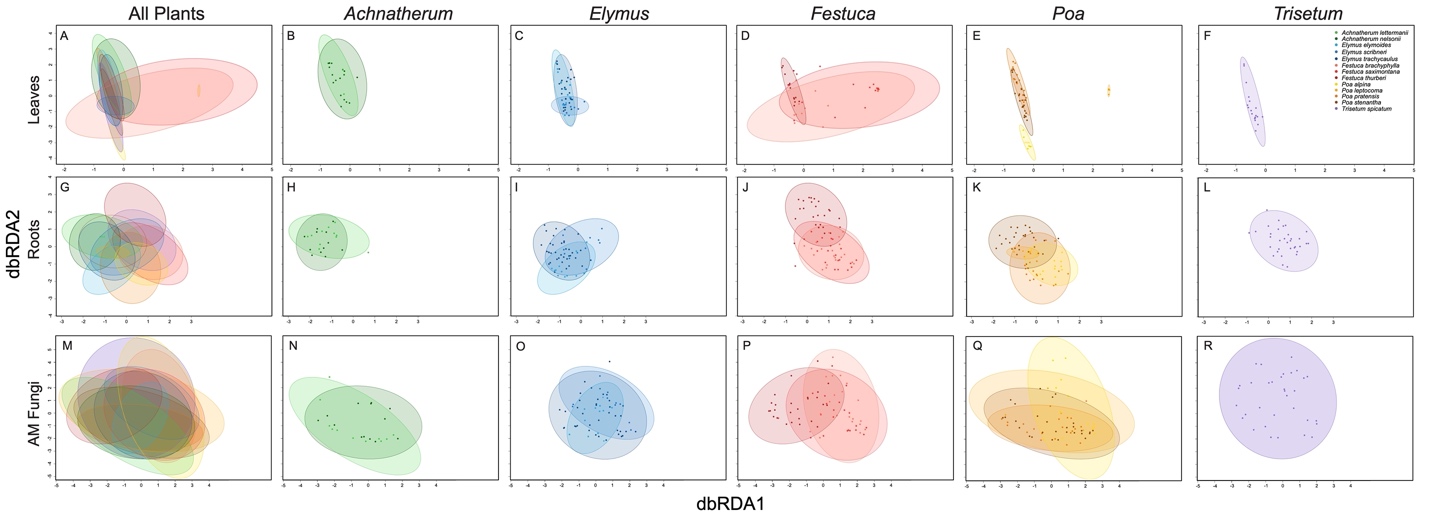

Supplement: Supplementary file 3 — Figure S3 [file 43705_2022_107_MOESM3_ESM.docx]
