# Supplementary material for: Grass species identity shapes communities of root and leaf fungi more than elevation
Source: ISME Commun. 2022 Mar 17;2:25. doi: 10.1038/s43705-022-00107-6 (PMC9723685; doi:10.1038/s43705-022-00107-6)
Supplement: Supplementary file 4 — Figure S4 [file 43705_2022_107_MOESM4_ESM.docx]

**Figure S4**. Abundance of pathogens (A,B), saprotrophs (C,D) and mutualists (E,F) in leaf (A,C,E) and root (B,D,F) fungal symbiont communities. Sequence reads were proportionally transformed to equalize sampling effort in each sample. Then sequence read abundances were summed for each functional group. Means and 95% confidence intervals are plotted for each grass species. Tukey posthoc designations after correcting for false discovery rate of alpha = 0.05 are denoted with lower case letters. AM fungi are not included as they are all designated mutualists by current fungal functional databases.
