# Supplementary material for: Grass species identity shapes communities of root and leaf fungi more than elevation
Source: ISME Commun. 2022 Mar 17;2:25. doi: 10.1038/s43705-022-00107-6 (PMC9723685; doi:10.1038/s43705-022-00107-6)
Supplement: Supplementary file 5 — Table S1 [file 43705_2022_107_MOESM5_ESM.docx]

**Table S1.** Environmental parameters and plant species at each sampling site. Plant species abbreviations: ACLE – *Achnatherum lettermannii*, ACNE – *Achnatherum nelsonii*, ELEL – *Elymus elymoides*, ELSC – *Elymus scribneri*, ELTR – *Elymus trachycaulus*, FEBR – *Festuca brachyphylla*, FESA – *Festuca saximontana*, FETH – *Festuca thurberii*, POAL – *Poa alpina*, POLE – *Poa leptocoma*, POPR – *Poa pratensis*, POST – *Poa stenantha*, TRSP – *Trisetum spicatum*.

|  |  |  |  |  |  |  |  |  |  |  |  |
| --- | --- | --- | --- | --- | --- | --- | --- | --- | --- | --- | --- |
| **Gradient** | **Elevation**  **(m)** | **Grass Species Sequenced**  **(Leaves)** | **Grass Species Sequenced**  **(Roots)** | **Grass Species Sequenced**  **(AM fungi)** | **Latitude** | **Longitude** | **MAP (mm)** | **MAT (°C)** | **Total N (μg/g)** | **Soil pH** | **Soil Moisture (%)** |
| Avery | 2711 | ACNE, ELEL, ELTR | ACNE, ELEL,  ELTR | ACNE, ELEL,  ELTR | 38.865 | -106.912 | 563 | 13.3 | 65 | 6.0 | 6.1 |
| Avery | 2812 | ACNE, FETH, POPR | ACNE, POPR | ACNE, POPR | 38.865 | -106.912 | 605 | 12.9 | 80 | 6.8 | 12.5 |
| Avery | 2896 | ACNE, ELTR, FESA, FETH | ACNE, ELTR,  FESA, FETH | ACNE, ELTR, FESA, FETH | 38.951 | -106.986 | 674 | 12.4 | 55 | 5.5 | 11.3 |
| Avery | 2982 | ELTR, FETH, POPR | ACNE, ELTR,  FETH, POPR | ACNE, ELTR, FETH, POPR | 38.962 | -106.985 | 711 | 12.0 | 63 | 5.7 | 12.3 |
| Avery | 3135 | FETH, POPR | FETH, POPR | FETH, POPR | 38.966 | -106.983 | 773 | 11.3 | 119 | 6.7 | 13.6 |
| Avery | 3192 | ELTR | ELTR | ELTR, POST | 38.971 | -106.984 | 798 | 11.0 | 139 | 6.2 | 13.0 |
| Avery | 3347 | ACLE, ELTR, FESA, FETH, POLE, POST, TRSP | ACLE, ELTR, FESA, FETH, POLE, POST, TRSP | ACLE, ELTR, FESA, FETH, POLE, TRSP | 38.975 | -106.978 | 861 | 10.2 | 11 | 6.6 | 9.6 |
| Avery | 3455 | ACLE, ELTR, FETH, POST | ACLE, ELTR,  FETH, POST | ACLE, ELTR, FETH, POST | 38.927 | -106.978 | 895 | 9.8 | 68 | 6.0 | 8.1 |
| Avery | 3655 | ELSC, FEBR, POAL, TRSP | ELSC, FEBR,  POAL, TRSP | ELSC, FEBR, POAL, TRSP | 38.984 | -106.970 | 987 | 8.8 | 17 | 5.0 | 19.7 |
| Cinnamon | 2749 | ACLE, ACNE, ELTR, FETH | ACNE | ACNE, ELTR | 38.882 | -106.962 | 593 | 13.2 | 132 | 6.0 | 10.1 |
| Cinnamon | 2799 | ACNE, ELTR, FESA, POPR | ACNE,  FESA, POPR | ACNE, ELTR, FESA, POPR | 38.897 | -106.979 | 621 | 12.9 | 104 | 5.9 | 14.1 |
| Cinnamon | 2932 | ACLE, ACNE, ELEL, ELTR, FESA, FETH, POLE, POPR, TRSP | ACLE, ELEL,  ELTR, FESA,  FETH, POLE,  POPR, TRSP | ACNE, ELEL, ELTR, FESA, FETH, POLE, POPR, TRSP | 38.935 | -107.011 | 690 | 12.3 | 29 | 5.9 | 16.4 |
| Cinnamon | 3025 | FETH, POST | POST | POST | 38.945 | -107.028 | 734 | 11.9 | 50 | 5.9 | 14.4 |
| Cinnamon | 3181 | ELTR, POST | ELTR, POST | ELTR, POST | 38.962 | -107.031 | 802 | 11.1 | 97 | 6.1 | 13.4 |
| Cinnamon | 3223 | ELTR, POST | POST | ELTR, POST | 38.962 | -107.049 | 821 | 10.9 | 64 | 6.1 | 12.5 |
| Cinnamon | 3366 | ELTR, FESA, POLE, POST, TRSP | ACLE, ELTR,  FESA, POLE,  POST, TRSP | ACLE, ELTR, FESA, POLE, POST, TRSP | 38.970 | -107.030 | 879 | 10.2 | 84 | 5.7 | 9.9 |
| Cinnamon | 3416 | FESA, POST, TRSP | FESA, POST,  TRSP | FESA, POST,  TRSP | 38.991 | -107.065 | 912 | 10.0 | 56 | 5.5 | 11.8 |
| Cinnamon | 3579 | FEBR, POST, TRSP | FEBR, POST,  TRSP | FEBR, POST,  TRSP | 38.994 | -107.068 | 979 | 9.2 | 13 | 4.6 | 21.1 |
| Cinnamon | 3665 | FEBR, POST, TRSP | FEBR, POST,  TRSP | FEBR, POST,  TRSP | 38.995 | -107.069 | 1015 | 8.8 | 34 | 5.1 | 12.0 |
| Cinnamon | 3726 | ELSC, FEBR, POAL, TRSP | ELSC, FEBR,  POAL, TRSP | ELSC, FEBR, POAL, TRSP | 38.995 | -107.070 | 1041 | 8.5 | 47 | 5.4 | 8.9 |
| Hunter’s Hill | 2824 | ACLE, ACNE, FETH, POPR | ACLE, ACNE,  FETH, POPR | ACLE, ACNE, FETH, POPR | 38.848 | -106.820 | 586 | 12.7 | 78 | 5.5 | 7.5 |
| Hunter’s Hill | 3060 | ELTR, FETH, POPR | ELTR, FETH,  POPR | ELTR, FETH, POPR | 38.904 | -106.784 | 686 | 11.5 | 2 | 5.6 | 10.1 |
| Hunter’s Hill | 3171 | FESA, FETH, POPR, TRSP | FESA, POPR,  TRSP | FESA, POPR,  TRSP | 38.926 | -106.778 | 735 | 11.0 | 22 | 6.4 | 12.8 |
| Hunter’s Hill | 3249 | ACLE, ELTR, FESA, FETH, TRSP | ACLE, ELTR,  FESA, FETH,  TRSP | ACLE, ELTR, FESA, FETH,  TRSP | 38.926 | -106.792 | 770 | 10.6 | 10 | 6.3 | 11.0 |
| Hunter’s Hill | 3322 | FESA, FETH,  POST, TRSP | FESA,  POST, TRSP | FESA,  POST, TRSP | 38.929 | -106.791 | 799 | 10.2 | 20 | 5.4 | 9.1 |
| Hunter’s Hill | 3416 | FESA, FETH, POST | ELTR, FESA,  POST | ELTR, FESA,  POST | 28.930 | -106.789 | 844 | 9.7 | 11 | 5.8 | 11.4 |
| Hunter’s Hill | 3531 | FESA, FETH, POPR | ELTR, FESA,  FETH, POPR | ELTR, FESA, FETH, POPR | 38.933 | -106.787 | 886 | 9.2 | 4 | 5.9 | 10.0 |
| Hunter’s Hill | 3629 | ELSC, ELTR, FEBR, FESA, FETH | ELSC, ELTR,  FEBR, FESA,  FETH, TRSP | ELSC, ELTR,  FEBR, FESA, FETH, TRSP | 38.938 | -106.787 | 927 | 8.8 | 29 | 6.2 | 13.6 |
| Hunter’s Hill | 3724 | ELSC, TRSP | ELSC, TRSP | ELSC, TRSP | 38.941 | -106.788 | 966 | 8.3 | 37 | 7.7 | 8.3 |
| Hunter’s Hill | 3827 | FEBR, POAL, TRSP | ELTR, FEBR,  POAL, TRSP | ELTR, FEBR, POAL, TRSP | 38.946 | -106.788 | 1009 | 7.8 | 2 | 6.8 | 18.7 |
| Ruby | 2822 | ACLE, ACNE, ELEL, ELTR, FETH, POPR | ACLE, ACNE,  ELEL, ELTR,  FETH, POPR | ACLE, ACNE, ELEL, ELTR,  FETH, POPR | 38.864 | -107.032 | 635 | 12.9 | 32 | 5.8 | 8.3 |
| Ruby | 2945 | ACLE, FESA, FETH, POPR, POST | ACLE, FESA,  FETH, POPR,  POST | ACLE, FESA, FETH, POPR, POST | 38.856 | -107.070 | 692 | 12.3 | 38 | 5.7 | 9.4 |
| Ruby | 3055 | ACLE, ELTR, POST | ACLE, ELTR,  POST | ACLE, ELTR,  POST | 38.864 | -107.106 | 747 | 11.9 | 41 | 5.8 | 19.1 |
| Ruby | 3128 | ACLE, POST | ACLE, POST | ACLE, POST | 38.874 | -107.106 | 779 | 11.5 | 93 | 5.4 | 14.8 |
| Ruby | 3199 | ACLE, ELTR, TRSP | ACLE, ELTR,  TRSP | ACLE, ELTR,  TRSP | 38.884 | -107.113 | 812 | 11.1 | 15 | 5.0 | 18.3 |
| Ruby | 3333 | ELTR, FESA, POST | ELTR, FESA,  POST | ELTR, FESA,  POST | 38.895 | -107.117 | 870 | 10.5 | 74 | 5.7 | 11.9 |
| Ruby | 3447 | FEBR, FESA, POST, TRSP | FEBR, FESA,  POST, TRSP | FEBR, FESA, POST, TRSP | 38.902 | -107.116 | 918 | 9.9 | 2 | 4.6 | 27.2 |
| Ruby | 3539 | ELSC, FEBR | ELSC, FEBR, TRSP | ELSC, FEBR, TRSP | 38.901 | -107.122 | 956 | 9.5 | 57 | 5.7 | 17.0 |
| Ruby | 3633 | ELSC, ELTR, FEBR, POAL, TRSP | ELSC, ELTR,  FEBR, POAL,  TRSP | ELSC, ELTR,  FEBR, POAL,  TRSP | 38.900 | -107.126 | 996 | 9.1 | 102 | 5.1 | 13.1 |
| Ruby | 3723 | ELSC, POAL, TRSP | ELSC, FEBR,  POAL, TRSP | ELSC, FEBR,  POAL, TRSP | 38.899 | -107.128 | 1032 | 8.6 | 12 | 5.7 | 20.0 |
| Ruby | 3833 | ELSC, ELTR, FEBR, TRSP | ELSC, ELTR,  FEBR, TRSP | ELSC, ELTR,  FEBR, TRSP | 38.897 | -107.128 | 1077 | 8.1 | 82 | 5.7 | 17.2 |
| Teocali | 2776 | ACNE, ELEL, ELTR, FETH, POPR | ACLE, ACNE,  ELEL, ELTR,  POPR | ACLE, ACNE, ELEL, ELTR,  FETH, POPR | 38.896 | -106.891 | 592 | 13.0 | 115 | 6.6 | 7.4 |
| Teocali | 2868 | FETH, POPR | FETH, POPR | FETH, POPR | 38.906 | -106.884 | 630 | 12.5 | 59 | 6.1 | 7.2 |
| Teocali | 2948 | ACNE, ELTR, FETH, POPR | ACNE, ELTR,  FETH, POPR | ACNE, ELTR, FETH, POPR | 38.929 | -106.878 | 666 | 12.1 | 13 | 6.5 | 16.1 |
| Teocali | 3047 | FETH, POPR, TRSP | FETH, POPR,  TRSP | FETH, POPR, TRSP | 38.944 | -106.887 | 712 | 11.6 | 75 | 6.6 | 11.9 |
| Teocali | 3157 | ELTR, FETH, POPR | ELTR, FETH,  POPR | ELTR, FETH, POPR | 38.948 | -106.890 | 758 | 11.1 | 85 | 6.0 | 9.6 |
| Teocali | 3275 | FETH, POST, TRSP | FETH, POST,  TRSP | ELTR, FETH, POST, TRSP | 38.948 | -106.881 | 804 | 10.5 | 42 | 6.6 | 11.6 |
| Teocali | 3351 | ELTR, FETH, POST | ELTR, FETH,  POST | FETH, POST | 38.947 | -106.878 | 835 | 10.2 | 90 | 6.2 | 9.1 |
| Teocali | 3443 | ELTR, FETH, POST, TRSP | ELTR, FETH,  POST, TRSP | ELTR, FETH, POST, TRSP | 38.950 | -106.876 | 873 | 9.7 | 114 | 6.2 | 7.3 |
| Teocali | 3553 | FETH, POST | ELTR, FETH,  POST | ELTR, FETH,  POST | 38.953 | -106.876 | 918 | 9.2 | 11 | 5.5 | 10.5 |
| Teocali | 3667 | FESA, FETH, POST, TRSP | FESA, FETH,  POST, TRSP | FESA, FETH, POST, TRSP | 38.956 | -106.878 | 966 | 8.7 | 42 | 6.1 | 11.7 |
| Teocali | 3771 | ELTR, FEBR, FESA, POAL, POST, TRSP | ELTR, FEBR,  FESA, POAL,  POST, TRSP | ELTR, FEBR, FESA, POAL, POST, TRSP | 38.952 | -106.878 | 1009 | 8.1 | 40 | 6.0 | 9.8 |
| Teocali | 3879 | ELSC, FEBR, POAL | ELSC, ELTR,  FEBR, POAL | ELSC, ELTR,  FEBR, POAL | 38.959 | -106.880 | 1054 | 7.6 | 8 | 5.1 | 23.5 |
| Teocali | 3954 | FEBR, POAL, TRSP | FEBR, POAL,  POST, TRSP | FEBR, POAL,  POST, TRSP | 38.960 | -106.883 | 1085 | 7.3 | 30 | 5.6 | 18.2 |
| Treasury | 2747 | ELTR, POPR | ELTR, FETH,  POPR | ELTR, FETH, POPR | 38.919 | -107.036 | 617 | 13.2 | 58 | 6.3 | 17.0 |
| Treasury | 2795 | ACNE, ELEL, ELTR, FETH, POPR, POLE, TRSP | ACNE, ELEL,  ELTR, FETH,  POPR, POLE,  TRSP | ACNE, ELEL,  ELTR, FETH,  POPR, POLE,  TRSP | 38.933 | -107.050 | 642 | 13.0 | 47 | 5.9 | 14.0 |
| Treasury | 2972 | POPR, POST, TRSP | POPR, POST,  TRSP | POPR, POST, TRSP | 38.973 | -107.062 | 726 | 12.1 | 48 | 6.2 | 12.9 |
| Treasury | 3038 | ELTR, POST | ACNE, ELTR,  POST | ACNE, ELTR, POST | 38.965 | -107.059 | 751 | 11.8 | 316 | 6.0 | 15.5 |
| Treasury | 3197 | ELTR, FESA, FETH, POPR, POST, TRSP | ELTR, FESA,  FETH, POPR,  POST, TRSP | ELTR, FESA,  FETH, POPR,  POST, TRSP | 38.971 | -107.059 | 842 | 10.7 | 8 | 6.0 | 15.7 |
| Treasury | 3257 | POST | POST | POST | 38.976 | -107.059 | 892 | 10.2 | 24 | 6.3 | 12.5 |
| Treasury | 3371 | POST | POST |  | 38.986 | -107.062 | 912 | 10.0 | 100 | 5.5 | 13.8 |
| Treasury | 3419 | FEBR, POST, TRSP | FEBR, POST,  TRSP | FEBR, POST,  TRSP | 38.988 | -107.065 | 912 | 10.0 | 16 | 5.3 | 13.7 |
| Treasury | 3521 | FEBR, POAL | FEBR, POAL | FEBR, POAL | 38.996 | -107.075 | 958 | 9.5 | 52 | 4.7 | 18.8 |
| Treasury | 3598 | ELSC, ELTR, FESA, TRSP | ELSC, ELTR,  FEBR, FESA,  TRSP | ELSC, ELTR,  FEBR, FESA, POAL, TRSP | 39.000 | -107.081 | 992 | 9.1 | 110 | 6.0 | 9.7 |
| Treasury | 3698 | FEBR, POAL | POAL | FEBR, POAL | 39.000 | -107.084 | 1033 | 8.6 | 96 | 6.6 | 10.9 |
| Treasury | 3819 | ELSC, ELTR, FEBR, FESA, POAL, TRSP |  |  | 39.006 | -107.091 | 1083 | 8.1 | 27 | 7.3 | 12.2 |
| Treasury | 4023 | FEBR, POAL, TRSP |  |  | 39.011 | -107.096 | 1171 | 7.1 | 2 | 5.5 | 13.3 |
