# Supplementary material for: Grass species identity shapes communities of root and leaf fungi more than elevation
Source: ISME Commun. 2022 Mar 17;2:25. doi: 10.1038/s43705-022-00107-6 (PMC9723685; doi:10.1038/s43705-022-00107-6)
Supplement: Supplementary file 6 — Table S2 [file 43705_2022_107_MOESM6_ESM.docx]

**Table S2**. Sequencing depth metrics for leaf endophytes, root endophytes, and AM fungi.

| **Guild** | **Average Sequencing Depth** | **Standard Deviation Sequencing Depth** | **Minimum Sequencing Depth** | **Maximum Sequencing Depth** | **Average # OTUs** | **Standard Deviation OTUs** | **Minimum # OTUs** | **Maximum #**  **OTUs** |
| --- | --- | --- | --- | --- | --- | --- | --- | --- |
| Leaf Endophytes | 1624 | 3062 | 50 | 27053 | 49 | 41 | 5 | 288 |
| Root Endophytes | 12518 | 12543 | 239 | 134893 | 135 | 63 | 21 | 418 |
| AM fungi | 8163 | 5235 | 448 | 58777 | 54 | 21 | 16 | 139 |
